# Adipokine profile in acute heart failure: associations with natriuretic response and prognosis

**DOI:** 10.1093/eschf/xvag150

**Published:** 2026-05-26

**Authors:** Jan Biegus, Rafał Tymków, Gracjan Iwanek, Robert Zymliński, Mateusz Guzik, Magdalena Hurkacz, Wojciech Sopyla, Piotr Gajewski, Piotr Ponikowski

**Affiliations:** Department of Cardiology, Clinical Department of Intensive Cardiac Care, Faculty of Medicine, Wroclaw Medical University, Institute of Heart Diseases, Borowska 213, Wroclaw 50-556, Poland; Department of Cardiology, Jan Mikulicz Radecki University Hospital in Wrocław, Borowska 213, Wroclaw 50-556, Poland; Department of Cardiology, Clinical Department of Intensive Cardiac Care, Faculty of Medicine, Wroclaw Medical University, Institute of Heart Diseases, Borowska 213, Wroclaw 50-556, Poland; Department of Cardiology, Jan Mikulicz Radecki University Hospital in Wrocław, Borowska 213, Wroclaw 50-556, Poland; Department of Cardiology, Clinical Department of Intensive Cardiac Care, Faculty of Medicine, Wroclaw Medical University, Institute of Heart Diseases, Borowska 213, Wroclaw 50-556, Poland; Department of Cardiology, Jan Mikulicz Radecki University Hospital in Wrocław, Borowska 213, Wroclaw 50-556, Poland; Department of Cardiology, Clinical Department of Intensive Cardiac Care, Faculty of Medicine, Wroclaw Medical University, Institute of Heart Diseases, Borowska 213, Wroclaw 50-556, Poland; Department of Cardiology, Jan Mikulicz Radecki University Hospital in Wrocław, Borowska 213, Wroclaw 50-556, Poland; Department of Cardiology, Clinical Department of Intensive Cardiac Care, Faculty of Medicine, Wroclaw Medical University, Institute of Heart Diseases, Borowska 213, Wroclaw 50-556, Poland; Department of Cardiology, Jan Mikulicz Radecki University Hospital in Wrocław, Borowska 213, Wroclaw 50-556, Poland; Department of Clinical Pharmacology, Faculty of Pharmacy, Wroclaw Medical University, 211A Borowska Street, Wroclaw 50-556, Poland; Department of Clinical Pharmacology, Faculty of Pharmacy, Wroclaw Medical University, 211A Borowska Street, Wroclaw 50-556, Poland; Department of Cardiology, Clinical Department of Intensive Cardiac Care, Faculty of Medicine, Wroclaw Medical University, Institute of Heart Diseases, Borowska 213, Wroclaw 50-556, Poland; Department of Cardiology, Jan Mikulicz Radecki University Hospital in Wrocław, Borowska 213, Wroclaw 50-556, Poland; Department of Cardiology, Clinical Department of Intensive Cardiac Care, Faculty of Medicine, Wroclaw Medical University, Institute of Heart Diseases, Borowska 213, Wroclaw 50-556, Poland; Department of Cardiology, Jan Mikulicz Radecki University Hospital in Wrocław, Borowska 213, Wroclaw 50-556, Poland

**Keywords:** Adipokines, Acute heart failure, Decongestion, Spot urine sodium, Prognosis

## Abstract

**Introduction:**

Adipose tissue signalling is linked to heart failure (HF), but its role in acute HF (AHF), especially concerning early diuretic response, is unclear. Early natriuretic response to loop diuretics is vital for effective decongestion.The objective was to characterize and evaluate the associations between circulating adipokine profiles, early natriuretic response, and clinical outcomes in AHF.

**Methods:**

We conducted a prospective observational study including 262 consecutive AHF patients. Early diuretic response was assessed by measuring post-diuretic spot urine sodium (uNa^+^) 2 h after intravenous loop diuretic administration. Associations between adipokines and uNa^+^ were assessed, and an adipokine score was constructed based on markers associated with uNa^+^. Clinical outcomes (all-cause mortality and a composite of death or HF hospitalization) were evaluated over 1 year using Cox regression models.

**Results:**

Patients with impaired natriuretic response (uNa^+^ < 90 mmol/L) exhibited higher levels of selected Domain-3 adipokines, including chemerin and FABP4. Individual adipokines showed modest associations with early natriuretic response. Visfatin was significantly associated with lower post-diuretic uNa^+^, while IL-6, FABP4, and Adipsin demonstrated directional but non-significant trends. An adipokine score integrating these selected adipokines was independently associated with uNa^+^ after multivariable adjustment (β 3.37, 95% CI 1.51–5.23; *P* < .001). During a 1-year follow-up, several adipokines: IL-6, resistin, and FABP4, were independently associated with mortality, while FABP4 consistently demonstrated associations with both impaired natriuresis and adverse outcomes.

**Conclusions:**

In AHF, individual adipokines are modestly associated with the early natriuretic response, whereas an integrated adipose-inflammatory profile shows a more consistent association with sodium excretion and adverse outcomes. These findings suggest that adipokine signalling may reflect biological pathways associated with diuretic response and prognosis, though the results should be considered hypothesis-generating.

## Graphical Abstract

**Graphical Abstract:**
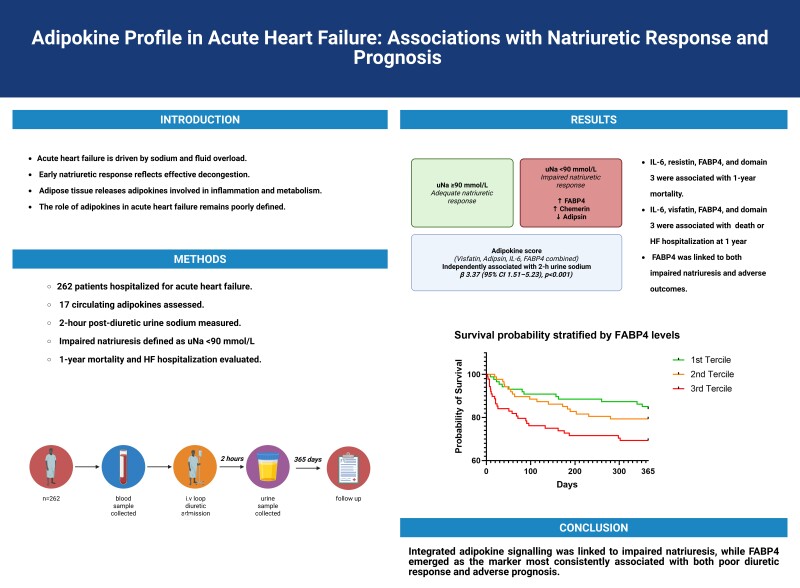
Adipose–inflammatory signalling is associated with impaired natriuresis and adverse outcomes in acute heart failure.

## Introduction

Adipose tissue is increasingly recognized as an active endocrine organ that modulates cardiovascular and renal function through the secretion of adipokines.^1,2^ These mediators have been implicated as central to the pathophysiology of heart failure with preserved ejection fraction (HFpEF), where they may promote systemic inflammation, endothelial dysfunction, and myocardial remodelling.^1^ Adipose tissue is also thought to influence total body water, promote plasma volume expansion, disrupt water–sodium balance, and impact the loop diuretic requirements.^3–5^

In contrast, the role of adipokines in acute heart failure (AHF) remains largely unexplored. Acute heart failure is characterized by considerable disturbances in sodium and fluid handling—phenomena universal for all HF types, irrespective of the ejection fraction, and the early natriuretic response to diuretic therapy is a critical determinant of decongestion and clinical outcomes.^6–8^ Several factors are linked to a reduced diuretic response in AHF, resulting in less effective decongestion, and adipose tissue, along with adipokines—the signalling molecules it produces—may serve as a mechanistic mediator of at least part of this adverse phenotype.^9,10^ However, whether adipokines modulate natriuresis and kidney function, thereby influencing the clinical course of AHF, remains unknown.

We hypothesized that adipokine signalling in AHF is not driven by isolated biomarkers but rather reflects integrated biological pathways that influence sodium handling and clinical outcomes (as potential modulators of sodium handling). Accordingly, the aim of the present study was to comprehensively characterize circulating adipokine profiles across the spectrum of AHF and to investigate their association with early natriuretic response and subsequent clinical outcomes.

## Methods

### Study design and population

This is an observational study based on a prospective registry of patients hospitalized for AHF at the Institute of Heart Diseases, Wroclaw Medical University. Consecutive patients admitted with a primary diagnosis of AHF were screened for inclusion, and those who provided written informed consent were enrolled as early as possible during hospitalization.

Acute HF was defined according to current guideline criteria, requiring the presence of symptoms and signs of congestion. Adult patients (≥18 years) were eligible for inclusion if they provided informed consent during the early phase of hospitalization. Key exclusion criteria comprised conditions that could confound the assessment of congestion or diuretic response, including cardiogenic shock, acute coronary syndrome, acute pulmonary embolism, and acute aortic syndromes.^11^ This study protocol was approved by the local Bioethics Committee, and all procedures were conducted in accordance with the principles of the Declaration of Helsinki.

### Study procedures

Upon enrolment, detailed clinical data were collected. Blood and urine samples were obtained according to a predefined protocol to characterize biochemical and urinary profiles. In addition, biological material was collected and stored for subsequent biomarker analyses, including assessment of adipokine profiles. Patients received standard-of-care treatment for AHF in our institution, including intravenous loop diuretics, as determined by the treating physician. The study was non-interventional, and no therapeutic decisions were influenced by the investigators.

### Assessment of diuretic response

Diuretic response: the early natriuretic response was assessed using post-diuretic urine sodium (uNa^+^) measurements obtained at predefined time points after initiation of IV loop diuretic therapy. The post-diuretic urine samples were collected 2 h after the loop diuretic exposure and assessed in a local laboratory. The loop diuretic dose was left to the treating physician's discretion, and it was recommended that current guidelines be followed.

Post-diuretic uNa^+^ was selected as the primary marker of diuretic response because it is an objective and pathophysiologically grounded measure of early natriuretic effectiveness. We did not rely on 24-h diuresis as the primary response metric, as it is substantially influenced by factors unrelated to intrinsic diuretic responsiveness, including fluid intake, inaccuracies in urine collection, temporal changes in treatment, and differences in diuretic dosing during the entire collection period. Because loop diuretic administration was not standardized in this study, 24-h urine output was considered less reliable and less informative than post-diuretic uNa^+^.

### Follow-up and outcomes

Patients were followed longitudinally after hospital discharge. Clinical outcomes included all-cause mortality and rehospitalization, which were analysed separately and in composite form. Follow-up data were obtained through systematic telephone contacts and review of available medical documentation.

### Adipokine assessments

Plasma concentrations of the target adipokines were measured using commercially available Enzyme-Linked Immunosorbent Assay (ELISA) kits, according to the manufacturers’ instructions. Interleukin-6 (IL-6; D6050B), tumour necrosis factor alpha (TNF-α; DTA00D), resistin (DRSN00), adiponectin (DRP300), chemerin (DCHM00), fatty acid-binding protein 4 (FABP4; DFBP40), leptin (DLP00), and retinol-binding protein 4 (RBP4; DRB400) were quantified using Human Quantikine ELISA kits (R&D Systems, Minneapolis, MN, USA). Adipsin (EHCFD) and apelin (EEL026) were measured using Invitrogen™ Human ELISA kits. Adipolin (E3692Hu) was assessed using a kit from the Bioassay Technology Laboratory. Asprosin (SEA332Hu) was measured using a Cloud-Clone ELISA kit. CTRP9 (orb1473485) was quantified using a Human CTRP9 ELISA kit (Biorbyt). Neprilysin (DL-NEP-Hu) was measured using a Human Neprilysin (NEP) ELISA kit (DLDevelop). Omentin-1 (RD191100200R) and visfatin (RAG004R) were determined using Human ELISA kits (BioVendor R&D Systems). Neuregulin (MBS054957) was assessed using a human ELISA kit (MyBioSource, Inc.). Optical density was measured at 450 nm using a microplate reader (TK Biotech), and a standard curve was generated for each assay. All analyses were performed in the Department of Clinical Pharmacology laboratory at Wroclaw Medical University.

### Statistical analysis

Continuous variables are presented as mean (standard deviation) or median (interquartile range), as appropriate, while categorical variables are expressed as counts and percentages. Due to their non-normal distribution, adipokine concentrations were log-transformed for analysis. Between-group comparisons were performed using Student’s *t*-test or the Mann–Whitney *U* test for continuous variables and the χ^2^ test for categorical variables. Given the lack of well-defined reference ranges for adipokines, their values were standardized to z-scores to facilitate comparison of deviations.

This analysis was exploratory. The adipokine score was derived from adipokines selected based on their univariable associations with post-diuretic uNa+. No formal correction for multiple testing was applied; accordingly, the findings should be interpreted as hypothesis-generating and warrant external validation.

Associations between adipokine profiles, natriuretic response, and clinical outcomes were evaluated using correlation analyses and regression models, as appropriate. Time-to-event analyses were performed using Cox proportional hazards models, with results expressed as hazard ratios (HRs) and 95% confidence intervals (CIs). For multivariable analyses examining the association between adipokines and clinical outcomes, only adipokines that showed a significant association with prognosis in a model including all measured adipokines were selected. Subsequently, each selected adipokine was entered individually into multivariable models adjusted for established prognostic factors, including age, baseline serum creatinine, serum sodium, systolic blood pressure, and NT-proBNP. A two-sided *P*-value <.05 was considered statistically significant. All analyses were conducted using standard statistical software.

## Results

### Baseline characteristics

A total of 262 AHF patients were included in the study. The majority of participants were male (*n* = 183, 69.9%), and the mean age was 68.2 ± 14.4 years. The population represented a broad spectrum of HF phenotypes, with a mean left ventricular ejection fraction of 37.5 ± 15.4%. HFpEF was diagnosed in *n* = 105 (40.1%) of patients. *De novo* AHF occurred in *n* = 89 (34%). The mean body mass index (BMI) was 30.6 ± 8.5 kg/m^2^, and the median NT-proBNP was 6886.7 (3746.0–12301.1) pg/ml. Detailed baseline characteristics are presented in *Table 1*.

**Table 1:** Baseline characteristics of patients with acute heart failure (*n* = 262)

Parameter	Population
Sex (male)	183 (69.85%)
Age (years)	68.15 ± 14.4
Heart rate (beat/min)	89.3 ± 23.6
Systolic blood pressure at admission (mmHg)	125.3 ± 24.2
Diastolic blood pressure at admission (mmHg)	77.2 ± 15.9
Left ventricle ejection fraction (%)	37.5 ± 15.4
HFpEF *n* (%)	105 (40.08%)
Acute heart failure (*de novo)*	89 (34%)
Ischaemic aetiology of heart failure:	86 (33%)
BMI	30.6 ± 8.5
Blood Count: Haemoglobin (g/dl)	12.7 ± 2.2
WBC (G/L)	8.2 ± 3.2
PLT (G/l)	228.1 ± 93.1
AST (IU/L)	29.0 [23.0–40.0]
ALT (IU/L)	25.5 [17.0–40.0]
Bilirubin (mg/dl)	1.6 ± 1.1
Na (mmol/l)	140.0 ± 4.8
Creatinine (mg/dl)	1.3 ± 0.6
BUN (mg/dl)	60.7 ± 34.6
hsCRP (mg/l)	10.2 [4.2–23.6]
NT-proBNP (pg/ml)	6886.7 [3746.0–12301.1]
Troponin I (ng/ml)	27.4 [15.5–62.3]
uNa^+^	98.68 ± 27.54
Hospitalization (days)	8 [5–12]
Lactate on admission (mmol/l)	1.5 ± 0.8

Data shown as *n* (%). Mean ± SD or median [Q25–Q75].

ALT, alanine aminotransferase; AST, aspartate aminotransferase; BUN, blood urea nitrogen; CRP, C reactive protein; Na, serum sodium; NT-proBNP, N-Terminal Pro-B–type natriuretic peptide; PLT, platelets; WBC, white blood cell.

### The adipokine profile in acute heart failure

Median adipokine levels varied across the study population, indicating substantial heterogeneity in adipose tissue-derived signalling among patients with AHF. The interpretation of absolute adipokine concentrations remains inherently limited because no well-established reference values have been proposed for this population and clinical setting. Although reference ranges for several markers are provided by assay manufacturers, these values should be regarded as assay-specific guidance rather than rigorously established normative ranges and should be interpreted with caution. Therefore, adipokine levels were additionally standardized using z-scores to enable analyses within the context of the study cohort. A detailed list of observed values and the suggested ranges is presented in *Table 2*.

**Table 2:** The adipokine profile in acute heart failure

Adipokine	Level	Suggested normal range^a^
Domain 1		
TNFa [pg/ml]	4 (4–4)	<15.6 pg/ml
Adipolin [ng/ml]	3.844 (2.944–5.072)	N/A^b^
Adiponectin [ng/ml]	11541.65 (6239.2–18 667.5)	1359–20 691 ng/ml
CTRP9 [ng/ml]	0.532 (0.264–0.831)	N/A^b^
Omentin-1 [ng/ml]	911.902 (440.337–1165.244)	356–666 ng/ml
Visfatin [ng/ml]	0.718 (0.327–1.714)	0.2–1.5 ng/ml
Neuregulin 4 [ng/ml]	3.311 (2.859–3.87)	N/A^b^
Domain 2		
Adipsin [µg/ml]	3.778 (2.472–5.793)	N/A^b^
Apelin [pg/ml]	1850.695 (37.5–3374.649)	121.5–298.7 pg/ml
Domain 3		
IL-6 [pg/ml]	9.119 (2.765–26.2)	<12.9 pg/ml
Resistin [ng/ml]	19.095 (14.601–28.886)	5.73–24.5 ng/ml
Asprosin [ng/ml]	1.284 (0.689–2.68)	N/A^b^
Chemerin [ng/ml]	85.946 (57.876–128.194)	60.1–199 ng/ml
FABP4 [ng/ml]	82.51 (53.78–201.05)	4.256–38.880 ng/ml
Leptin [pg/ml]	5454.85 (2791.5–14428.7)	men: 2205–11149 pg/ml women: 3877–77273 pg/ml
Neprilysin [ng/ml]	5.747 (2.075–13.724)	N/A^b^
RBP4 [ng/ml]	41205.674 (33333.333–51312.057)	12 200–43 000 ng/ml

^a^Based on the ELISA test kit manufacturer.

^b^Data not provided by the manufacturer.

Against this background, several adipokines showed consistent directional differences relative to the suggested reference intervals (*Table 2*). Within Domain 1, most of the adipokines were within the suggested ranges; only Omentin-1 was above the suggested threshold. In Domain 2, Apelin levels were generally higher relative to the available reference values. In Domain 3, RBP4 and FABP4 were above the suggested reference values.

### Comparison of adipokines between good vs insufficient diuretic response

Patients were stratified based on the early (2 h) post-diuretic urinary sodium (uNa^+^) with a threshold of 90 mmol/L. There were 71 (33.65%) patients with uNa^+^ < 90 mmol/l. Baseline characteristics were largely similar between groups, including age, sex, blood pressure, and left ventricular ejection fraction (all *P* > .05). Patients with insufficient natriuretic response had higher NT-proBNP: 9173.4 [3888.5–18865.6] vs 6368.7 [3568.2–10278.1] pg/ml, serum creatinine 1.5 ± 0.7 vs 1.2 ± 0.5 mg/dl, and BUN 76 ± 44 vs 52.4 ± 23.5 mg/dl at baseline compared to the group with uNa^+^ ≥ 90 mmol/l, respectively (all *P* < .05) (*Table 3*).

**Table 3:** Comparison of patients in different populations by diuretic response

Variables:	uNa < 90	uNa ≥ 90	*P*
Sex (male)	49 (69.0%)	100 (71.4%)	.718
Age (years)	67.4 ± 15.6	68 ± 13.9	.775
Heart rate (beat/min)	86.2 ± 23.9	91.2 ± 21.5	.139
Systolic blood pressure at admission (mmHg)	122.4 ± 22.7	125.7 ± 23.3	.35
Diastolic blood pressure at admission (mmHg)	79.2 ± 15.6	76.3 ± 16.8	.258
Left ventricle ejection fraction (%)	36.5 ± 16.9	38.2 ± 15.1	.444
HFpEF n (%)	25 (35.2%)	61 (43.6%)	.245
Acute heart failure (*de novo)*	17 (23.9%)	60 (42.9%)	.007
Ischaemic aetiology of heart failure:	17 (23.9%)	50 (35.7%)	.083
BMI	29.6 ± 5.6	31.2 ± 10.3	.268
Blood count: Haemoglobin (g/dl)	12.5 ± 2.4	12.8 ± 2	.247
WBC (G/L)	8.3 ± 3.9	7.9 ± 2.8	.489
PLT (G/l)	228 ± 105.4	230.4 ± 91	.873
AST (IU/L)	32 [23–40]	28 [23–39]	.759
ALT (IU/L)	27.5 [17–48]	25 [16–39]	.617
Bilirubin (mg/dl)	1.4 ± 0.8	1.6 ± 1	.335
Na (mmol/L)	139 ± 7.2	140.7 ± 3.5	.030
Creatinine (mg/dl)	1.5 ± 0.7	1.2 ± 0.5	<.001
BUN (mg/dl)	76 ± 44	52.4 ± 23.5	<.001
hsCRP (mg/l)	10.5 [4.7–24.9]	9.6 [4.3–19.6]	.971
NT-proBNP (pg/ml)	9173.4 [3888.5–18865.6]	6368.7 [3568.2–10278.1]	.002
Troponin I (ng/ml)	23.7 [14.4–60.8]	27.4 [16–57.4]	.453
**Adipokines**			
TNFa [pg/ml]	4 [4–4]	4 [4–4]	.376
Adipolin [ng/ml]	3.5 [2.8–4.8]	3.8 [2.9–5.2]	.348
Adiponectin [ng/ml]	10698.3 [6483–15627.7]	12023.2 [6594–20389.4]	.135
CTRP9 [ng/ml]	0.5 [0.2–0.8]	0.5 [0.3–0.8]	.251
Omentin-1 [ng/ml]	990.9 [386.3–1402.9]	855.4 [440.3–1141.6]	.299
Visfatin [ng/ml]	0.8 [0.3–2.4]	0.7 [0.3–1.7]	.279
Neuregulin 4 [ng/ml]	3.3 [2.9–3.8]	3.3 [2.8–3.9]	.923
Adipsin [µg/ml]	3.4 [2.3–5.1]	3.7 [2.5–5.9]	.032
Apelin [pg/ml]	1298.2 [37.5–3374.6]	1895 [37.5–3362.8]	.726
IL-6 [pg/ml]	11.5 [2.9–32.6]	10.2 [4.1–24.8]	.568
Resistin [ng/ml]	18 [14.2–26.5]	18.6 [14.3–25.7]	.967
Asprosin [ng/ml]	1.2 [0.6–2.2]	1.4 [0.7–2.8]	.169
Chemerin [ng/ml]	107.3 [72.2–144.4]	83.7 [57.9–125.7]	.024
FABP4 [ng/ml]	107.64 [52.9–503.7]	75.95 [51.8–159.6]	.005
Leptin [pg/ml]	5595.6 [2791.5–13708.4]	5229.5 [2508.4–14242]	.506
Neprilysin [ng/ml]	3.9 [1.7–11.9]	6.3 [2.2–14.5]	.236
RBP4 [ng/ml]	42021.3 [37789.1–53652.5]	39471.8 [30602.8–49894.4]	.237

Patients with insufficient natriuretic response had lower Adipsin 3.4 [2.3–5.1] vs 3.7 [2.5–5.9] µg/ml (*P* < .03), and higher adipokines from domain 3: Chemerin 107.3 [72.2–144.4] vs 83.7 [57.9–125.7] ng/ml, (*P* = .024), and FABP4 107.6 [52.9–503.7] vs 75.9 [51.8–159.6] ng/ml (*P* = .005), compared to the group with higher post-diuretic uNa^+^ (*Table 3*).

### Adipokines and their associations with post-diuretic natriuretic response

In a regression model, four adipokines were identified as potential predictors of post-diuretic uNa^+^ in AHF. Visfatin was significantly associated with post-diuretic uNa^+^ with beta coefficient (β) (95% CI): −20.32 (−34.52; −6.12), *P* = .005. There was also a trend for a significant association with Adipsin β (95% CI): 16.45 (−0.51; 33.41) *P* = .057; IL-6 [pg/ml] β (95% CI): −5.82 (−12.23; 0.59) *P* = .075, and FABP4 β (95% CI): −8.31 (−17.28; 0.67), *P* = .070. The z-score of domain 3 was significantly associated with β (95% CI): −1.26 (−2.50; −0.01), *P* = .049 (*Table 4*).

**Table 4:** Circulating adipokines as predictors of post-diuretic uNa^+^ in acute heart failure

Variables	b (95% CI)	*P*
TNFa [pg/ml]	−14.50 (−37.76 to 8.76)	.221
Adipolin [ng/ml]	1.67 (−11.57 to 14.91)	.805
Adiponectin [ng/ml]	3.51 (−4.00 to 11.03)	.360
CTRP9 [ng/ml]	14.86 (−16.94 to 46.66)	.360
Omentin-1 [ng/ml]	−3.72 (−11.76 to 4.31)	.364
Visfatin [ng/ml]	−20.32 (−34.52 to −6.12)	.005
Neuregulin 4 [ng/ml]	−32.49 (−80.98 to 16.01)	.189
Adipsin [µg/ml]	16.45 (−0.51 to 33.41)	.057
Apelin [pg/ml]	0.58 (−3.21 to 4.37)	.76
IL-6 [pg/ml]	−5.82 (−12.23 to 0.59)	.075
Resistin [ng/ml]	−10.15 (−26.40 to 6.10)	.221
Asprosin [ng/ml]	8.80 (−7.80 to 25.41)	.299
Chemerin [ng/ml]	−1.18 (−11.85 to 9.49)	.828
FABP4 [ng/ml]	−8.31 (−17.28 to 0.67)	.070
Leptin [pg/ml]	−0.07 (−6.32 to 6.19)	.983
Neprilysin [ng/ml]	3.17 (−5.04 to 11.39)	.449
RBP4 [ng/ml]	−4.15 (−10.19 to 1.89)	.178
Z-score domain 1	−0.00 (−0.01 to 0.01)	.368
Z-score domain 2	1.79 (−0.84 to 4.43)	.182
Z-score domain 3	−1.26 (−2.50 to −0.01)	.049

### Association of adipokine score with post-diuretic natriuretic response

Given that the association with natriuresis did not appear to be driven by any single adipokine, we constructed an adipokine score integrating those adipokines that showed the strongest univariable associations with post-diuretic urinary sodium (uNa^+^): Visfatin, Adipsin, IL-6, and FABP4. The resulting Adipokine Score demonstrated a strong association with post-diuretic natriuretic response β (95% CI): 3.46 (1.72–5.20), *P* < .001. Importantly, in a multivariable model adjusted for age, serum creatinine, serum sodium, NT-proBNP, and systolic blood pressure, the Adipokine Score remained independently associated with post-diuretic uNa β (95% CI): 3.37 (1.51–5.23), *P* < .001.

### The impact of adipokines on outcomes

**Figure 1:**
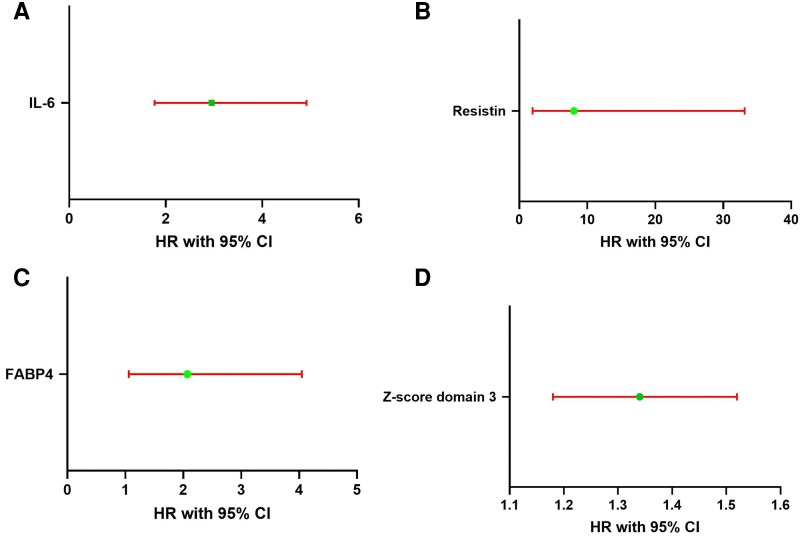
Associations between selected adipokines and 1-year mortality in multivariable Cox regression models. Results are presented as hazard ratios (HRs) with 95% confidence intervals. *A*, IL-6. *B*, Resistin. *C*, FABP4. *D*, Z-score domain 3. Adjusted for age, baseline serum creatinine, serum sodium, systolic blood pressure, and NT-proBNP

**Figure 2:**
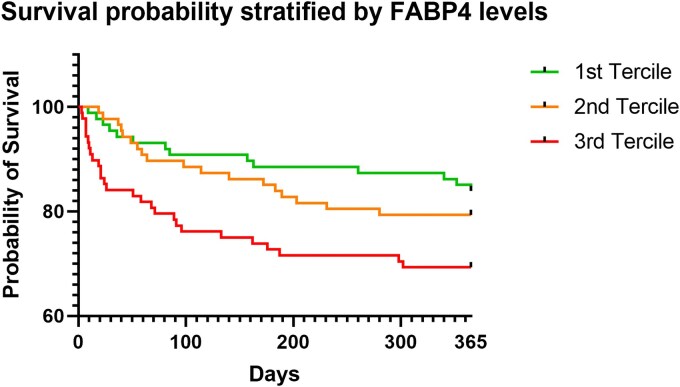
Kaplan–Meier curves for 1-year survival by FABP4 tertiles

There were 59 deaths (22.5%), and 130 patients (49.6%) reached the composite endpoint of death or HF hospitalization during the 365-day follow-up. In multivariable analysis, IL-6 (HR 2.95, 95% CI 1.77–4.92), resistin (HR 8.04, 95% CI 1.95–33.14), FABP4 (HR 2.07, 95% CI 1.06–4.05), and the z-score of domain 3 (HR 1.34, 95% CI 1.18–1.52) were independently associated with 1-year mortality, all *P* < .05 (*Table 5*, and *Figure 1A–D*). For the composite endpoint, visfatin (HR 0.24, 95% CI 0.08–0.72), IL-6 (HR 1.72, 95% CI 1.22–2.43), FABP4 (HR 2.07, 95% CI 1.06–4.05), and the z-score of domain 3 (HR 1.13, 95% CI 1.04–1.23) remained significantly associated with outcome, all *P* < .05 (*Table 6*). The survival by the FABP4 tertiles is presented in *Figure 2*.

**Table 5:** Prognostic significance of adipokines in AHF (univariable and multivariable model)

Univariable				Multivariable^a^		
Variables	χ^2^	HR (95% CI)	*P*	χ^2^	HR (95% CI)	*P*
TNFa (log)	0.25	0.60 (0.08–4.45)	.613			
Adipolin (log)	2.55	1.92 (0.86–4.26)	.11			
Adiponectin (log)	2.95	2.08 (0.90–4.78)	.086			
CTRP9 (log)	2.13	4.62 (0.59–35.90)	.144			
Omentin-1 (log)	0.08	0.92 (0.53–1.61)	.775			
Visfatin (log)	0.21	0.77 (0.26–2.32)	.643			
Neuregulin 4 (log)	1.96	8.27 (0.43–158.51)	.161			
Adipsin (log)	2.81	2.58 (0.85–7.81)	.093			
Apelin (log)	1.12	1.16 (0.88–1.52)	.289			
IL-6 (log)	23.1	2.98 (1.91–4.66)	<.001	17.25	2.95 (1.77–4.92)	<.001
Resistin (log)	19.4	14.45 (4.40–47.38)	<.001	8.31	8.04 (1.95–33.14)	.004
Asprosin (log)	0.72	1.61 (0.54–4.86)	.396			
Chemerin (log)	0.05	0.92 (0.46–1.84)	.821			
FABP4 (log)	7.30	2.38 (1.27–4.46)	.007	4.50	2.07 (1.06–4.05)	.034
Leptin (log)	3.24	0.70 (0.48–1.03)	.071			
Neprilysin (log)	1.70	1.47 (0.82–2.62)	.191			
RBP4 (log)	1.20	1.76 (0.64–4.82)	.273			
Z-score domain 1 (log)	2.93	1.08 (0.99–1.19)	.087			
Z-score domain 2 (log)	3.73	1.19 (1.00–1.42)	.053			
Z-score domain 3 (log)	22.6	1.29 (1.16–1.43)	<.001	19.89	1.34 (1.18–1.52)	<.001

Death during a 365-day period.

^a^Adjusted for: age, serum creatinine, serum Na^+^, systolic blood pressure, NT-proBNP.

**Table 6:** Prognostic significance of adipokines in AHF (univariable and multivariable model)

Univariable				Multivariable^a^		
Variables	χ^2^	HR (95% CI)	*P*	χ^2^	HR (95% CI)	*P*
TNFa (log)	0.53	0.59 (0.14–2.44)	.467			
Adipolin (log)	0.01	1.02 (0.56–1.88)	.943			
Adiponectin (log)	0.53	1.16 (0.78–1.71)	.466			
CTRP9 (log)	0.25	0.68 (0.15–3.06)	.614			
Omentin-1 (log)	5.26	0.66 (0.46–0.94)	.022	1.276933	0.729 (0.421–1.262)	.258
Visfatin (log)	4.94	0.41 (0.18–0.90)	.026	6.469013	0.24 (0.08–0.72)	.011
Neuregulin 4 (log)	0.01	0.94 (0.11–8.03)	.956			
Adipsin (log)	1.55	1.63 (0.76–3.51)	.213			
Apelin (log)	0.02	1.01 (0.85–1.21)	.890			
IL-6 (log)	10.27	1.63 (1.21–2.20)	.001	9.390324	1.72 (1.22–2.43)	.002
Resistin (log)	2.27	1.82 (0.84–3.96)	.132			
Asprosin (log)	0.72	0.72 (0.33–1.54)	.395			
Chemerin (log)	2.70	1.58 (0.92–2.71)	.100			
FABP4 (log)	5.08	1.62 (1.06–2.45)	.024	4.50131	2.07 (1.06–4.05)	.034
Leptin (log)	0.08	0.96 (0.73–1.27)	.769			
Neprilysin (log)	0.30	0.90 (0.62–1.31)	.583			
RBP4 (log)	0.68	1.18 (0.80–1.73)	.406			
Z-score domain 1 (log)	3.06	0.95 (0.89–1.01)	.080			
Z-score domain 2 (log)	0.93	1.06 (0.94–1.20)	.336			
Z-score domain 3 (log)	7.28	1.09 (1.02–1.17)	.007	8.347708	1.13 (1.04–1.23)	.004

Rehospitalization and death during a 365-day period.

^a^Adjusted for: age, serum creatinine, serum Na^+^, systolic blood pressure, NT-proBNP.

## Discussion

In this analysis of AHF patients, we provide a comprehensive characterization of circulating adipokine profiles across the full spectrum of AHF, irrespective of left ventricular ejection fraction. Several key findings emerge from this study.

Firstly, the association between individual adipokines and the natriuretic response, assessed by post-diuretic urinary sodium excretion, was modest overall. Only selected adipokines (visfatin, adipsin, IL-6, and FABP4), particularly those clustered in biological domain 3, were associated with impaired natriuresis (post-diuretic 2 h urine Na^+^).^1^ This is biologically plausible, as natriuretic response in AHF is governed by complex interconnected mechanisms, including renal perfusion, neurohormonal activation, tubular function, and interstitial congestion.^9,10,12–14^ Therefore, it was unlikely that a single adipokine would exert a dominant effect, and the relationships among several molecules and their domains may better reflect the modulation of the diuretic response.

In this context, a more integrated signal emerged at the level of the adipose tissue–inflammation axis.^15^ As no robust normative data are available (see above), we standardized the adipokine concentrations using z-scores. Thus, activation of domain 3 as a whole, as captured by standardized (z-score) analyses, was associated with a less favourable natriuretic response. Further to this end, a cluster of adipokines, defined by our adipokine score that integrated signals from four adipokines (visfatin, adipsin, IL-6, and FABP4), was independently associated with diuretic response. This suggests that groups of adipokines and their interactions may jointly influence sodium regulation in AHF rather than the overactivation of a single one. However, this observation is hypothesis-generating and requires further evaluation. Our results support the concept that composite biological pathways (inflammatory-adipose signalling), rather than individual biomarkers, may better reflect the mechanisms underlying diuretic response in HF. Importantly, this hypothesis is biologically plausible as inflammatory signals might be associated with decongestion.^16–19^ Moreover, in the recent SUMMIT trial, the use of Tirzepatide in the HFpEF population resulted in a lower risk of the primary composite endpoint that consisted of worsening HF events.^20^ Therefore, the observed modulation of circulatory volume-pressure overload and systemic inflammation in the study may at least in part be explained by the adipokine-natriuresis interaction.^21^ Taken together, these observations support a hypothesis in which adipose tissue dysfunction contributes to the pathophysiology of AHF not through isolated pathways, but via complex, interconnected biological networks.^22,23^ Further studies are warranted to validate these findings and to determine whether adipokine-guided approaches may help refine risk stratification, identify patients at risk of diuretic resistance, or serve as a therapeutic target for more effective decongestion.

Moreover, we observed that the overall adipokine profile was broadly comparable between AHF patients with HFpEF and HFrEF. This finding is noteworthy given the suggested link between adipose tissue function and the HFpEF phenotype. Our data suggest that, in the acute setting, adipose tissue–related signalling may represent a shared pathophysiological component across the AHF spectrum rather than a mechanism restricted to preserved ejection fraction only. Given that visceral adipose tissue is present and metabolically active across the EF spectrum, its endocrine effects are unlikely to be EF-specific, a finding reflected in our cohort.^2^

Several adipokines showed associations with outcomes; however, consistent independent associations were primarily observed for IL-6, resistin, FABP4, and the domain 3 score. These findings indicate that adipokine signalling may carry prognostic information beyond traditional risk markers in AHF. Importantly, these associations persisted after adjustment for established prognostic factors, suggesting that adipokines may capture distinct aspects of disease biology, potentially related to inflammation, metabolic dysregulation, and organ crosstalk.

Among the evaluated adipokines, FABP4 was the only marker consistently associated with both an impaired natriuretic response and worse clinical outcomes, including increased mortality. This finding suggests that FABP4 may reflect a pathophysiological axis linking congestion, renal dysfunction, and metabolic inflammation and thus may serve as an integrative biomarker of the cardio–renal–metabolic interplay in AHF. However, our study needs to be interpreted with caution and treated as a hypothesis-generating. Taken together, our findings suggest that integrated adipose–inflammatory signalling, rather than individual biomarkers, may better capture the biological substrate of impaired natriuretic response in AHF.

In conclusion, in AHF, individual adipokines show only modest associations with natriuretic response, whereas the inflammatory–adiposity cluster, represented by domain 3 or the adipokine score, demonstrates a more consistent relationship with impaired natriuresis. Independent associations with adverse prognosis were observed, particularly for IL-6 and FABP4. FABP4 was the only marker consistently associated with both impaired natriuretic response and adverse clinical outcomes. These findings should be considered hypothesis-generating.

### Limitations

This study has several limitations. First, it was conducted at a single European centre, which may limit the generalizability of the findings. Second, the analyses should be considered hypothesis-generating, particularly given the broad panel of adipokines evaluated. Third, the adipokine score was developed and assessed within the same cohort and was not externally validated; therefore, its reproducibility and clinical applicability require confirmation in independent populations. Moreover, the observational design precludes any conclusions regarding mechanistic causality, and the identified associations should not be interpreted as evidence of direct biological effects of individual adipokines on natriuresis or outcomes. Finally, natriuretic response in AHF is inherently variable and influenced by multiple dynamic factors; therefore, modest effect sizes at the level of single biomarkers were expected, and the observed associations should be interpreted within the context of this biological and clinical variability.

## Declarations

### Data Availability

The data that support the findings of this study are available from the corresponding author upon reasonable request.

### Pre-registered Clinical Trial Number

None supplied.
